# How to harness the effects of exudates and microbes that support beneficial plant–plant interactions for sustainable agriculture

**DOI:** 10.1371/journal.pbio.3003416

**Published:** 2025-10-16

**Authors:** Eva Marina Stirnemann, Joëlle Sasse

**Affiliations:** 1 Institute of Plant and Microbial Biology, University of Zurich, Zurich, Switzerland; 2 Molecular Agrar and Plant Ecology, Agroscope, Zurich, Switzerland

## Abstract

Root exudates, species-specific metabolites released by plants into the rhizosphere, shape plant nutrient uptake, plant–plant and plant–microbiome interactions. When optimized, such interactions boost the productivity of sustainable agricultural systems such as intercropping and crop rotation. However, why certain combinations of crops are beneficial and others are not, remains unclear. This Essay outlines how optimal crop combinations could be determined, focusing on the molecular interplay of crops and their microbial partners. With an advanced understanding of root exudation and its effects on microbes, new strategies for sustainable farming could be unlocked, reducing reliance on fertilizers and pesticides, and tackling challenges raised by a changing climate.

## Abstract 2

Intercropping and crop rotation are ancient agricultural practices that increase yield and disease resistance in crops. This Essay discusses the role of plant-derived metabolites and plant-associated microbes in these agricultural systems and how they can contribute to sustainable agriculture.

## Introduction

Before intensive agricultural practices were introduced, agriculture relied on low-input systems to optimize yield. Farmers recognized the central importance of plant–plant and plant–soil interactions and identified strategies to optimize productivity by trial-and-error. Spatial strategies such as intercropping [1,2], in which multiple crop species such as grasses and legumes are co-cultivated, as well as temporal strategies such as crop rotation [3,4], where multiple crop species are grown in an alternating manner, were developed and employed successfully for centuries. In the past century, the Green Revolution introduced intensive agriculture based on the input of fertilizer and pesticides to maintain productivity, and the old strategies were mostly abandoned in favor of efficient monoculture settings. However, with diminishing resources and a changing climate, new strategies to combat abiotic and biotic stresses in crops are warranted, and even resource-intensive regions are looking into the mechanisms that made older agricultural systems successful, to translate these into their newer systems [1,5]. In this Essay, we focus on the examples of intercropping and crop rotation and their underlying molecular mechanisms to determine what makes these ancient systems successful.

A general hypothesis that can be formed is that wild crop relatives accustomed to low nutrient levels rely on the optimization of interactions with beneficial partners to cope with stresses. Many of these traits were not selected in modern cultivars used in intensive agricultural settings because genetic diversity was reduced and production relied on input of fertilizers and pesticides, rendering beneficial interactions less important. Breeding was predominantly focused on aboveground traits such as yield or resistance to pathogens, and selection of belowground traits such as the development of a specific root architecture and interactions with soil organisms were, at most, indirectly in focus [6,7]. This impacted the ability of modern cultivars to interact with beneficial partners such as mycorrhiza and rhizobia [8]. Modern cultivars also generally feature reduced microbial diversity associated with roots [9]. Thus, to improve plant-beneficial interactions for sustainable agriculture, factors driving these associations need to be revealed in modern cultivars and compared to wild ancestors.

To understand the driving factors for plant–microbe interactions, their molecular mechanisms must first be revealed. Root exudates are one such mechanism: they comprise soluble compounds that are released from roots into the rhizosphere, the soil surrounding the root. Root exudates comprise a wide variety of chemicals, ranging from low-molecular weight compounds such as amino acids and sugars to high-molecular weight compounds such as flavonoids and coumarins (for reviews on exudate composition, consider [10–12]). Importantly, although exudate profiles share some similarities across plant species [13], there are also distinctions between species or cultivars [14–18]. Furthermore, exudates change based on the developmental stage of the plant [19,20], environmental factors [21], and the presence of abiotic and biotic stresses. For example, drought increases exudation of organic acids and secondary metabolites [21–25], and pathogen presence can trigger the release of antimicrobial compounds, such as camalexins and glucosinolates in the model dicot *Arabidopsis thaliana* treated with elicitors (molecules triggering plant defense mechanisms), or aromatic organic acids in barley challenged with *Fusarium* [21,26,27].

Exudates have various roles in soil, acting as food and signals to soil-dwelling microorganisms such as bacteria and fungi, as well as to macroorganisms such as nematodes and other plants [10,21]. Such plant–organism interactions are driven by changes in plant metabolism and exudation [20,28–30]; for example, plant–plant interactions impact exudation and microbiomes [31], and exudation in turn affects the composition of the microbial community [17,32]. Aromatic organic acids structure the root-associated community, as they are only metabolized by rhizo-competent bacteria [20]. For further information on how exudation is impacted by external factors such as the environment and on how exudation influences microbiomes, see [10,21,33–35]. Agriculturally relevant examples of plant–microbe interactions shaped by exudates are crop–arbuscular mycorrhizal fungi (hereinafter, mycorrhiza) and legume–rhizobia symbioses that increase phosphate and nitrogen uptake, respectively [36,37]. Both symbioses are initiated by increasing the exudation of signaling compounds in nutrient-deficient conditions and rely on intricate signaling pathways and nutrient exchanges [38]. When challenged with pathogens, plants ‘cry for help’ by altering the composition of exudates to attract beneficial microbes that help with defense [39,40]. Furthermore, exuded signals such as strigolactones and benzoxazinoids mediate plant–plant interactions that range from avoidance to attraction [41,42].

Overall, the optimal interaction of plant roots with soil-residing organisms poses many advantages for the plant. Engineering these interactions in an agricultural setting would enable a reduction in fertilizer and pesticide input for a more sustainable agriculture. For this to happen, the molecular mechanisms of plant–plant interactions, including exudation and interactions with microbes, need to be understood. Many recent studies and reviews suggest that we lack a systematic understanding of root exudation dynamics across cultivars and species, and across growth stages and conditions [10,43]. As a consequence, we do not know how most beneficial microbes are attracted to roots, thereby impeding the development of biological products for fertilization and protection [44–46]. In this Essay, we discuss the current state of knowledge on the molecular mechanisms that make intercropping and crop rotation setups successful. We describe the role of nitrogen-fixing bacteria, mycorrhizal fungi, and the general microbial community in beneficial plant–plant interactions, and how plant-derived exudates shape the functioning of these communities, exploring how a deeper understanding of root exudation and plant–microbe relationships can improve plant resilience to the biotic and abiotic stresses threatening agriculture.

## Plant–plant interactions in intercropping and crop rotation

**Fig 1:**
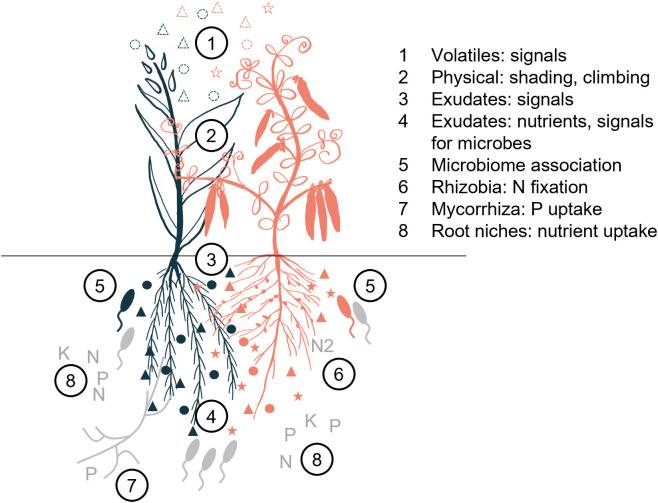
Aboveground and belowground plant–plant interactions. Interactions between a cereal (blue) and a legume (red) are conveyed by metabolites (circles, triangles, stars) or microbes (rod-shaped bacteria with flagella). General interactions with microbes or nutrients are indicated in gray, specific interactions are depicted in the respective colors.

When plants grow in a community with members of the same or different species, they interact both above and below ground. Aboveground, growth is shaped by shading, and the exchange of gaseous molecules conveys signals [47,48]. Belowground, root morphologies define accessibility and competition for resources, and signals are exchanged via metabolites or indirectly via changes in associated communities of, e.g., bacteria and fungi (Fig 1) [41,42]. Plant–plant interactions can be positive or negative, increasing or depressing growth and yield. Especially negative interactions have been well characterized in the literature regarding phenotypic changes and molecules inhibiting, e.g., growth of neighbors (allelochemicals). Recent reviews suggest that exudates and microbes also have a role, but that the effects of specific exudates, microbial strains, and the interplay with the specific environment remain rather unclear [41,49–51]. In the following sections, we focus on the molecular mechanisms of specific spatial or temporal beneficial interactions between plants.

### Spatial diversity increases crop resilience and productivity

**Fig 2:**
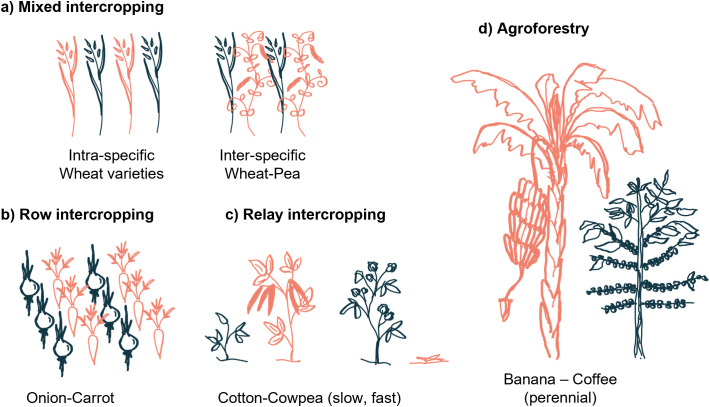
Different types of intercropping. Intercropped plants can belong to the same (a) or different (b–d) species. They can grow fully mixed (a) or rows (b). Many intercropped plants feature similar generation times (a, b), others are sown and harvested with different timing (c, d). Agroforestry (d) is an extreme case where trees are interspersed with shorter living crops.

Intercropping can have various beneficial effects, including increased yield quantity and quality with improved nutrient content, improved stress resilience, and lower risk for yield loss compared to monocultures [52]. However, intercropping setups in intensive agriculture face numerous challenges such as the synchronization of harvesting times of the crops and the separation of the fruits [53,54]. Various versions of intercropping exist on a spatial and temporal scale (Fig 2) [54]. Mixtures are often used for cereals, whereas alternating rows are chosen for different species. Intercropped plants can be sown at the same time, consecutively with partial overlap of the growth cycle (relay intercropping), or with different timing such as in agroforestry, where trees are interspersed with plants with shorter lifespans [52]. Crops in these systems interact distinctly on a spatial and temporal level. Thus, the underlying mechanisms resulting in beneficial growth and yield effects differ.

When multiple varieties or species are growing together, genetic diversity is increased, reducing overall pathogen pressure due to wider spacing of suitable hosts [55]. Furthermore, spatial niche differentiation above and below ground optimizes resource use; aboveground, optimal light usage and soil cover minimize soil erosion, evaporation, and weed growth [56,57], and belowground, distinct root systems explore different areas of the soil, reducing competition for locally available nutrients. A similar effect is observed in relay intercropping, where niches are differentiated temporally [58]. Niches can also be generated by differentiation of resources, for example, by the uptake of nitrate or ammonium as a nitrogen source. The effects of niche differentiation increase as higher numbers of distinct plant species are combined.

**Fig 3:**
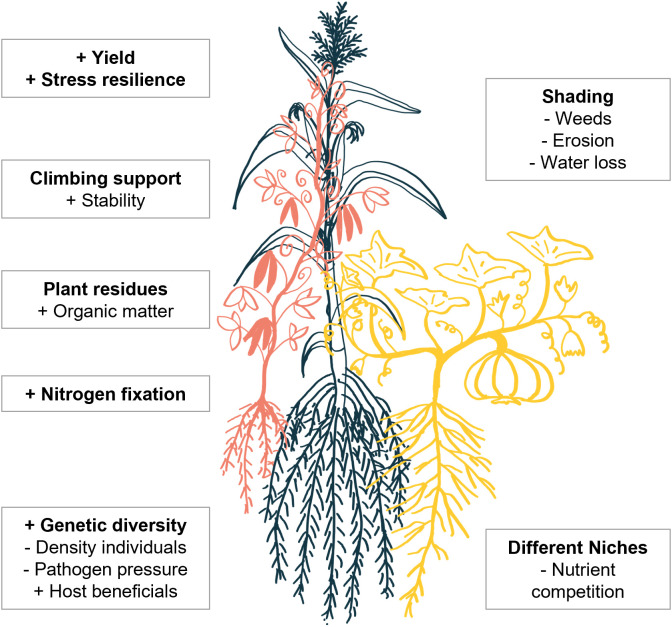
Three sisters intercropping system. Maize (blue), beans (red), and squash (yellow) are grown together. Factors making the system more robust and higher yielding than monocropping are listed in boxes.

One ancient, successful example of intercropping originates from central Mexico: co-cultivation of the “three sisters”—maize, beans, and squash [2]. Maize provides structural support, beans provide nitrogen due to their interaction with nitrogen-fixing rhizobia, and the ground dwelling squash limits the growth of weeds and water evaporation from the soil (Fig 3). Spatial niche differentiation is observed aboveground by different growth and leaf morphologies, and belowground with different vertical distribution of roots in the system, likely reflecting different nutrient acquisition strategies [59].

Maize yield per plant is generally comparable between monoculture and this intercropping system, whereas the yield gain of bean and squash depends on the nutrient content of the soil [59,60]. Although the three sisters have long been recognized as a key intercropping system, surprisingly few studies have focused on the molecular mechanisms underlying its success. Maize grown with beans and squash featured an adapted metabolic profile with more benzoxazinoid defense compound production and an altered volatile profile. The altered metabolism lowered damage caused by maize corn borer and increased the presence of pathogen predators [61]. Effects on metabolism of the other intercropped partners, on interactions with beneficial microbes, and on changes in soil physiochemical and biological parameters remain understudied. However, some insights have been generated on the molecular basis of other successful intercropping systems. Some of these examples are discussed in the following sections.

#### The importance of legumes and nitrogen fixation.

Most intercropping systems, including the three sisters, are based on legume–cereal combinations [62]. Due to the legume–rhizobia association, more nitrogen is also provided to the cereal, which is important especially in nutrient-poor soils. With this system, cereal yield and grain protein concentration can be increased [53]. In addition, intercropping legumes usually does not diminish yield of the other crop [54]. Beneficial effects of legumes on other crops are well researched and have been discussed elsewhere [63].

Importantly, however, legumes themselves are also shaped by intercropping. For example, the amount and activity of nodules is often affected in intercropping versus monocultures; in peanut intercropped with maize, flavonoids and coumarins were increased in peanut exudates. These compounds increased nodulating gene expression in nitrogen-fixing bacteria and boosted nodulation levels of *Bradyrhizobium*, increasing the amount of nodulation in intercropped peanut [64]. Interestingly, increased nodulation stimulation can also take place cross-species; in intercropped faba bean and maize, maize exudates stimulated nodulation and atmospheric N_2_ fixation in the beans. Remarkably, exudates of wheat or barley did not stimulate nodulation, showcasing species-specificity. The observed effect was not merely caused by nutrient supplementation by exudates, as wheat exudates boosted rhizobial growth, but maize exudates did not. The rhizobial signaling compound genistein was elevated in exudates, possibly explaining the observed effect [65]. Other studies, however, have found reduced nodule numbers but increased nodule weight and increased exudation of flavonoids by the legume partner in a relay intercropped soybean and maize system [66]. Thus, the nitrogen content of legumes and associated crops cannot directly be linked to nodule number, nodule size, or microbial activity.

It recently became clear that intercropped partners generally feature broad changes in exudation and microbiome composition, suggesting that distinct morphologies of intercropped plants are not only caused by changes in flavonoid–rhizobia interactions. In the peanut–maize study mentioned above, over 10% of exuded metabolites changed in abundance, and the microbiome was altered broadly, with increased growth and N-fixation activity of rhizobia [64]. In intercropped maize with soybean, microbiome diversity and connectivity were increased, including genes involved in soil nitrogen cycling [67]. Thus, a common feature of the studies examined is increased exudation of signals (flavonoids) for nitrogen-fixing bacteria, whereas effects on nodule number, size, and activity were diverse. Furthermore, broad changes in exudation and microbiome composition have been observed, with currently unclear effects on plant phenotypes and performance. It becomes evident that in cereal–legume intercropping systems, the plant partners influence each other directly by altering exudation and recognition of metabolites, and indirectly by changing the composition and activity of associated communities of organisms. Much remains to be done to disentangle the complex interactions of legume intercropping systems, accounting for the variety of nitrogen-fixing bacteria, other associated microbes, and investigating species-specificity of these interactions.

#### Beneficial effects of mycorrhizal inoculation depend on intercropped species.

Aside from nitrogen-fixing bacteria, mycorrhiza are prominent beneficial partners of many crops, improving phosphate and water uptake, and biotic stress resistance [68]. In intercropping systems, mycorrhization levels are often increased, although effects can vary widely depending on the intercropped partners. For example, mycorrhization levels increased in faba bean and in wheat when intercropped, with positive effects on root biomass of both species. Nitrogen fixation increased in faba bean, nitrogen transfer to wheat was improved, and nutrient levels in wheat increased [69]. Also in coriander intercropped with soybean, mycorrhization increased the macronutrient and micronutrient content, and changed the composition of essential oils [70]. By contrast, in wheat–lentil intercropping, mycorrhizal diversity and community structure changed depending on the plant species and year, but mycorrhization levels remained constant [71]. For intercropped tomato, an increase in mycorrhization was only observed when intercropped with leek, but not with other crops such as cucumber, basil, or fennel, illustrating the species-specificity of mycorrhization in intercropped systems [72]. Furthermore, intercropped systems inoculated with both mycorrhiza and rhizobia can have additive effects, as shown for faba bean and wheat [73].

Distinct mycorrhization in intercropped versus monocropped systems can be a consequence of altered exudation or microbiome composition; the plant hosts might exude different signals or nutrients, and/or the soil microbe community structure or activity could be altered. Altered exudation and microbiome profiles have been characterized for intercropped soybean and maize [74]; exudation was mostly reduced in maize, whereas in soybean, a complex picture with increased and decreased exudates was found. Specifically, exudation of tartaric, oxalic, aspartic, and malic acid, as well as alanine, was correlated with mycorrhization levels, but also with increased availability of nitrogen and phosphorus in soil and with a lowered soil pH [74]. As this study illustrates, changes in exudation and microbiome profiles are likely to be drivers not only of association with nitrogen-fixing bacteria as described above, but also of other microbial associations. Studying the underlying mechanisms of microbial associations in intercropped systems might help to resolve the contrasting phenotypes observed across different intercropped partners and environments.

#### Complex microbiome and exudation changes underlie observed crop phenotypes.

Microbial diversity is generally higher in intercropping versus monocropping systems, as observed for bacteria and fungi in soybean–sugarcane [75], maize–soybean and maize–potato [76] mixtures, and in mixtures of two or four intercropped plant species [77]. This increased diversity generally correlates with lower stochastic assembly of communities and increased network complexity [76], and with improved soil enzyme and ecosystem functions [75,76]. A few microbiome members exert beneficial functions, for example, a *Pseudomonas* strain in maize–peanut intercropping secretes a siderophore chelating iron, resulting in improved iron nutrition in peanut [78]. Although significant shifts in bacterial communities are observed in many studies, other studies identify more prominent responses in fungal communities. For example, for maize and the legume *Desmodium*, intercropped soils featured richer and more diverse fungal communities enriched with beneficial functions for intercropped versus monocropped setups [79].

Composition of plant-associated microbiomes is affected by the respective host [29,30]. Thus, it is not surprising that microbial community shifts in intercropping are likewise driven by the respective intercropped partners, as observed for maize intercropped with sesame, peanut, soybean, or sweet potato [80], or for various maize and bean cultivars [81]. Changes in microbial communities are shaped by changes in exudation. In intercropped maize, higher diversity in exudation and microbial community composition is indeed correlated [82]. The main exudates changing in intercropped maize (soyasapogenol B, 6-hydroxynicotinic acid, lycorine, shikimic acid, and phosphocreatine) were sufficient to reproduce the increased biomass and nutrient content of maize intercropping when added to natural soil. Importantly, the effect was abolished when the soil was sterilized, highlighting that changes in exudation shaped the microbial community that resulted in the observed phenotype [82]. In a tea–bean intercropping system, arabinofuranose was identified as a central metabolite structuring metabolite and microbial changes [83]. In this study, topsoil and subsoil samples were taken and distinct exudate and microbiome responses were observed, illustrating the importance of spatially resolved sampling [83]. Also, the developmental stage of crops was identified as a central factor shaping exudation in intercropped systems, as determined for sugarcane–peanut intercropping [84]. In this study, exudation of organic acids, sugars, and amino acids changed with development and intercropping status in both partners. Fumaric acid was identified as a central metabolite exuded by peanut that impacted activities of enzymes in the rhizosphere, resulting in improved soil nutrient levels [84].

Overall, these recent studies indicate that bacterial and fungal diversity is higher in intercropped versus monocropped systems. Increased diversity correlated with beneficial microbial functions, improved soil health, increased yield, and with improved ecosystem functions. Furthermore, increased diversity correlated with changes in exudation and microbiome composition and function, highlighting the importance of studying the interplay of crop metabolism and phenotypes with soil biotic and abiotic factors. General mechanisms on how exudation is affected by plant genotype, how specific changes in exudation affect microbial community structure, and how this impacts intercropped plants remain to be revealed.

#### The complex mechanisms for increased disease resistance.

Intercropping systems can not only boost biomass and yield but can also suppress weeds, insects and other pathogens [54]. For example, nematode damage to the focal crop is reduced by 40% in intercropping versus monocropping systems [85]. However, disease severity varies widely across studies, with some intercropped plant species reducing disease but others actually promoting infections [86]. Thus, it is critical to identify factors that are predictive of the success of specific intercropping combinations in a given environment.

Recently, the first studies have begun to unravel the molecular mechanisms underlying disease resistance in intercropping systems. In tomato intercropped with potato onion (a type of perennial onion), higher resistance against *Verticillium* wilt was detected. Potato onion increased exudation of the flavonoid taxifolin, which increased *Bacillus sp* recruitment to the tomato rhizosphere, induced tomato defense responses, and lowered disease [87]. The same intercropping system also increased tomato resistance to *Fusarium* wilt. This resistance was mediated by a different mechanism, involving increased degradation of the pathogen’s cell wall by soil enzymes, with the released cell wall components stimulating plant immunity [88]. Additional factors might contribute to the success of this system, as soil bacterial and fungal diversity was enhanced in this intercropping system [89], soil phosphorus availability was increased [90], and mycorrhizal abundances were increased in tomato and reduced in potato onion [91]. Also, in tomato intercropped with leek, *Fusarium* disease resistance was detected, but not in association with other crops [72]. This intercropping combination also increased mycorrhization (see above). Thus, disease resistance might directly or indirectly be mediated by mycorrhization. As a side note, mycorrhizal fungi might also be involved in the ‘cry for help’ mechanism; the success of mycorrhizal inoculation in agriculture is more strongly correlated with the presence of pathogenic fungi in fields than with nutrient deficiencies [92]. More methods by which metabolites and microbes can cause or avoid disease are discussed below for crop rotation systems. At present, we conclude that intercropping can increase disease resistance by various direct and indirect mechanisms. Changes in exudation, soil enzyme activity, and the presence of microbes can directly impact the presence or virulence of pathogens, or indirectly stimulate plant defenses or improve nutrient status, making the crop less susceptible. It is likely that a variety of mechanisms underlying disease resistance in intercropping systems remain to be discovered.

### Temporal diversity: crop rotation maintains ecosystem functions and yield

Crop rotation is a means to diversify plant species grown on a temporal rather than spatial scale. Like intercropping, crop rotation dates back centuries and is based on the observation that growth of the same crop for multiple seasons lowers yield. In ancient Mesopotamia, winter cereals were grown for 2 years, then rotated with a legume [3]. The Romans refined and spread this technique as the three-field system in medieval Europe, including a fallow field every 3 years for recovery [4]. In the 18th century, the four-field system became popular (wheat, turnips, barley, and clover), reducing the fallow time to one quarter. Later, a variety of rotations were implemented. During the Green Revolution, crop rotations were largely discontinued in countries that switched to intensive agricultural systems, where application of fertilizers and pesticides ensured high soil nutrient levels and pathogen resistance. However, crop rotation is still important globally, supporting plant and ecosystem health in diverse agricultural systems. Also, crop rotation will likely re-gain importance due to efforts moving towards a more sustainable agriculture with reduced inputs [5].

#### Important players in rotation schemes.

Similar factors to those discussed for intercropping define the success of crop rotation, and crop rotation causes similar beneficial effects. Crop rotation can increase crop yield, improve soil conditions, increase the presence of beneficial partners, disrupt weed and pathogen life cycles, decrease the release of greenhouse gases, and diminish the impact of abiotic and biotic stress [5]. A meta-study in China revealed yield increases of between 10% and 38% (average of 20%) when comparing crop rotations to continuous monocultures [93].

Crop rotation schemes have been mostly established by trial-and-error, with the exception of legumes: their presence in a rotation was recognized as important early on, as for intercropping systems discussed above. Legumes were added as green fertilizer to elevate soil nitrogen levels following the growth of cereals [5]. On average, rotations including legumes yielded 14% more compared to rotations without legumes [94]. Importantly, inclusion of legumes in the rotation needs to be balanced with other crops, as legume yields decline when grown in continuous monoculture; soybean yields declined over a 16-year period in monocropping setups, but remained high when rotated with other crops in 2- or 3-year schemes [94]. Rotations with corn and winter wheat resulted in the highest soybean yield and in the largest positive impact on soil health compared with monocropping or rotations with red clover, another legume [94]. The factors determining good crop rotation schemes and the underlying molecular mechanisms remain mostly unknown. However, one study focusing on peanut–cotton rotations found increased diversity of beneficial soil microbes that limited soil nutrient loss in rotations [95].

#### Disease-suppressive microbes in crop rotation.

Continuous monocropping depletes soils of nutrients and often results in poor soil structure. Moreover, it lowers microbial diversity in soils, depleting beneficial microorganisms [96]. This favors the accumulation of pathogens, rendering the soil disease-conducive. Legumes are especially prone to yield decreases when grown continuously. This is coupled with a decline in arbuscular mycorrhizal fungi and total soil microbial biomass and activity [97]. Continuously grown legumes are prone to infections with pathogenic fungi and nematodes, developing root rot, which reduces grain yield significantly. Consequentially, no legumes can be grown on such infested fields for up to 10 years [97]. For faba bean, this phenomenon was investigated on a molecular scale; continuous cropping lead to accumulation of the auto-toxin benzoic acid in soil. This lowered exudation of phenolic and flavonoid defense compounds and of enzyme activities in the rhizosphere, making faba bean more susceptible to *Fusarium* wilt. Measures such as intercropping or crop rotation reduced levels of benzoic acid, reducing disease susceptibility [98].

Diversifying crops by rotation alters metabolite input into the soil, the composition of the soil microbial community and the physiochemical soil structure. Life cycles of pathogens are interrupted, lowering pathogen pressure for the crop [99]. Soil-derived microbes can suppress disease by two mechanisms. General disease-suppressiveness is caused by the microbial community competing with pathogens for resources or niches and can confer resistance against a broad range of pathogens. This mode of resistance cannot be transferred between soils and is supported by the addition of organic material to soils to increase microbial diversity and activity [100]. By contrast, specific disease suppression is caused by selected microbes that interfere with a specific pathogen. This type of suppressiveness can be transferred between soils by transferring the microbe of interest [100]. The mechanisms causing specific disease resistance can partially overlap with mechanisms of general disease resistance, as beneficial microbes can also compete with the pathogen for resources. Further mechanisms are induction of immune responses in plants or inhibition of pathogen growth with enzymes or antibiotic production. Identification of specific disease-suppressive mechanisms is of interest to develop alternative strategies for combatting disease in agriculture. Examples of disease suppression by microbes are discussed excellently in a recent review [100]. Here, we focus on examples illustrating the interplay of disease, microbes and root exudation in crop rotations.

Even in intensive agricultural settings, crop rotations are central for the management of some pathogens. Take-all disease of wheat is caused by a soil-borne fungus. Despite being researched as the most important wheat disease for decades, crop rotation remains the most efficient strategy in controlling this disease [100,101]. Insights into molecular mechanisms were gained when it was noticed that fields infected with the disease somewhat recovered after a period of monocropping. In these fields, a specific *Pseudomonas* strain was identified that produces antimicrobial compounds inhibiting the growth of the pathogen. These bacteria conferring specific disease resistance are currently applied as a bioprotectant on an agricultural scale, together with seed-coating microbes that compete for the same niche as the pathogen in a general disease-suppressive mechanism [101]. Furthermore, cultivar-specific mechanisms were discovered; cultivars supporting low *Pseudomonas* richness supported lower take-all levels for the next generation grown in this field. Despite a few studies that have suggested that other microbes might be involved in combatting take-all disease, the molecular mechanisms remain rather unclear [102]. Open questions on how wheat or barley attract beneficial *Pseudomonas*, how high levels of beneficial microbes are being maintained on root surfaces, and how beneficial microbes and the pathogen interact with the microbiome remain to be resolved.

Another example of crop rotation reducing disease is *Fusarium* disease, which remained lower in peanut rotated with maize, potato, or soybean and coincided with changes in microbiome structure and function. Microbiota of soils from crop rotations inhibited *Fusarium* growth on plates more efficiently than microbiota of monocropped soils. Specific key microbes with reduced abundance in monocropped soils could be re-introduced into disease-infested soils and reduced disease severity [103]. The microbiome also mediated disease resistance in tomato against *Fusarium*. Susceptible and resistant cultivars feature distinct microbiome composition and distinct recruitment of disease-suppressive microbes in response to *Fusarium*-produced fusaric acid [104].

Also for potato, *Rhizoctonia* disease incidence was lower in 3-year rotations compared to monocropping. When grown in rotation with canola, barley, or sweet corn, potato tuber quantity and quality were high [105]. This correlated with changes in the microbial activity in the soil, the amount of culturable bacteria, and the diversity of microbial substrate use, especially of carbohydrates, carboxylic acids and amino acids. Also, these rotations exhibited increased mycorrhizal levels [105] and distinct fungal communities were present in suppressive versus conducive soils [106]. Specific microbes, such as Gamma-Proteobacteria [107] and specific *Bacillus* strains [108] are being identified as suppressing disease. However, the specific mode of interplay between the host, pathogen, and beneficial microbes in a given soil remains to be characterized.

In general, high crop diversity in the rotation scheme enhances the abundance of beneficial microbes. For example, incorporating Indian mustard and wild rocket into a rotation increased the presence of a specific *Pseudomonas* spp., which produced the antifungal compound 2,4-diacetylphloroglucinol, protecting the following cucumber crop from *Fusarium* wilt disease [109]. Including cover crops (crops grown primarily for soil protection rather than for yield) in the rotation further contributed to an improved nutrient balance and soil structure, counteracting the development of disease-conducive soils [96]. In addition to choosing an optimal crop rotation strategy, the soil can be treated by introducing a metabolite of choice (e.g., biofumigation); for example, *Brassicaceae* plants rich in glucosinolate defense compounds can be grown and incorporated into the soil before the crop of choice is grown on the field [110]. With this technique, yield could be increased by 30% due to lower abundance of pests and disease incidents.

In summary, crop rotation can suppress disease via general and specific mechanisms. Generally, increased microbial diversity allows competition with different pathogens for resources and niches. Specifically, altered exudation of plants triggered by pathogen presence can attract bioprotectant strains that compete with the pathogen via various mechanisms. As the latter defense mechanism relies on a specific microbial strain, this mechanism can be transferred between fields. However, it relies on an intricate understanding of the communication between crop and bioprotectant strain, and the requirements of the strain regarding nutrition, niche suitability and its incorporation into a preexisting microbial community, which warrants further studies of molecular mechanisms for most disease-suppressive systems.

## Conclusions and future perspectives

**Fig 4:**
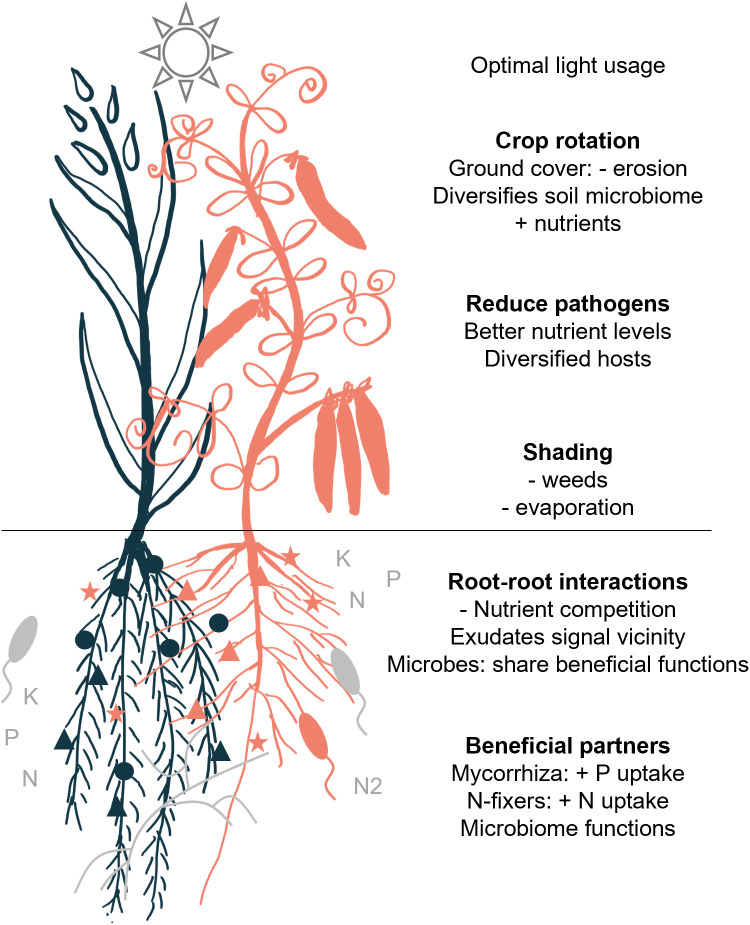
Beneficial effects of intercropping. Beneficial effects observed in intercropping of a cereal (blue) and a legume (red).

Growth of multiple plant species or cultivars on a spatial (intercropping) or temporal (crop rotation) level can result in multiple beneficial effects (Fig 4). In an optimal setup, multiple plant species optimize light usage, ground cover to reduce erosion, evaporation and weed growth, and add physical support. Belowground, different root morphologies allow for niche differentiation and the use of different nutrients.

The importance of legumes is well recognized, as their interaction with rhizobia results in higher nitrogen levels for associated crops. Interestingly, legume metabolism and associations with microbes are in turn shaped by their neighbors. Mycorrhizal associations also shift in intercropping versus monocropping systems, but general effects on mycorrhizal diversity and efficiency of the symbiosis remain to be elucidated. Furthermore, exudation of plants in intercropping systems is altered, leading to changes in microbe diversity and composition. Multiple exuded compounds and microbes improve stress resistance and growth in multi-species setups but so far, the key exudates and microbes identified seem to be system-specific. Mechanisms for increased disease resistance include changes in pathogen establishment, enzyme or signaling compound secretion, or the presence and activity of pathogen antagonists. Crucially, few of the observed beneficial effects seem to be caused by a single metabolite or microbe, but rather by a mixture of compounds or a community of microbes. This makes the characterization of mechanisms and the transfer of these effects from one environment to another difficult. Importantly, changes induced in soil microbiomes as well as in other abiotic and biotic soil factors can be long-lasting, shaping plant–soil interactions for multiple generations.

To make intercropping a feasible strategy to be implemented in modern agriculture, several important questions need to be answered (Box 1) and challenges need to be solved. First, straightforward procedures for seeding out, treating, and harvesting intercropped crops need to be developed. This includes adjusting agricultural equipment to be able to work in a row intercropping setting or to separate the fruits of two distinct crops after harvest. Software and subsidies will need adjustments for intercropping planning and must consider the additional workload.

**Box 1. Open questions.**

- What are the factors that make an intercropping or crop rotation setup (e.g., a cereal with a legume) beneficial in some cases but not in others? Can specific exuded compounds and microbes be identified that are common across intercropping systems? Are specific signals exchanged between the plants?
- Are there specific exudate–microbe interaction pairs that could be transferred between fields to make crops more resilient, even in monocropping setups?
- What exudates and microbial associations that increase stress resistance are present in wild ancestors but lacking in modern cultivars? Can they be re-introduced while balancing trade-offs with other desirable traits?
- What are the beneficial compounds and/or microbes that should be enriched in a soil to boost the growth of a specific crop?
- Why do legumes exude more signaling molecules for nitrogen-fixing bacteria when intercropped with a grass? How can this be leveraged for increased production?
- Why are some setups protective of disease but others conducive? Does intercropping or crop rotation promote the adaptation of pathogens to new hosts?

Besides these practical adjustments, more research is warranted to identify common signals, nutrients, and microbial functions that result in the desired effects of improved plant growth and yield. The interplay of plant metabolism and exudation on the microbial community, including nitrogen-fixing bacteria and mycorrhiza, pathogens and beneficial microbes, as well as on neighboring plants and on plants in the next generation is key to determine optimal intercropping and crop rotation schemes. Importantly, plant–plant and plant–microbe interactions are context specific, thus, abiotic factors need to be accounted for when predicting the success of different systems for a given environment. Genetic determinants underlying desirable traits in plant–microbe interactions could be identified using multi-omics techniques to connect genotype with phenotype. Furthermore, studying the interactions of roots with microbial communities of reduced complexity (SynComs) and using imaging techniques to explore spatial aspects of plant–microbe interactions will enhance our ability to engineer these interactions. Employing these techniques and the gained knowledge successfully will optimize plant stress resistance and yield while lowering inputs of fertilizers and pesticides in agricultural settings. An increased understanding of beneficial interactions will also allow us to engineer new approaches for systems that are well-adapted to novel biotic and abiotic stresses caused by a changing climate, or to maintain or restore soil health.
